# GNAS and KRAS Mutations are Common in Intraductal Papillary Neoplasms of the Bile Duct

**DOI:** 10.1371/journal.pone.0081706

**Published:** 2013-12-02

**Authors:** Motoko Sasaki, Takashi Matsubara, Takeo Nitta, Yasunori Sato, Yasuni Nakanuma

**Affiliations:** Department of Human Pathology, Kanazawa University Graduate School of Medicine, Kanazawa, Japan; West German Cancer Center, Germany

## Abstract

Intraductal papillary neoplasms of the bile duct (IPNB) shows favorable prognosis and is regarded as a biliary counterpart of intraductal papillary mucinous neoplasm (IPMN) of the pancreas. Although activating point mutations of GNAS at codon 201 have been detected in approximately two thirds of IPMNs of the pancreas, there have been few studies on GNAS mutations in IPNBs. This study investigates the status of GNAS and KRAS mutations and their association with clinicopathological factors in IPNBs. We examined the status of GNAS mutation at codon 201 and KRAS mutation at codon 12&13, degree of mucin production and immunohistochemical expressions of MUC mucin core proteins in 29 patients (M/F = 15/14) with IPNB in intrahepatic and perihilar bile ducts (perihilar IPNB) and 6 patients (M/F = 5/1) with IPNB in distal bile ducts (distal IPNB). GNAS mutations and KRAS mutations were detected in 50% and 46.2% of IPNBs, respectively. There was no significant correlation between the status of GNAS mutation and clinicopathological factors in IPNBs, whereas, the status of KRAS mutation was significantly inversely correlated with the degree of MUC2 expression in IPNBs (p<0.05). All IPNBs with GNAS mutation only showed high-mucin production. Degree of mucin production was significantly higher in perihilar IPNBs than distal IPNBs (p<0.05). MUC2 and MUC5AC expression was significantly higher in IPNBs with high-mucin production than those with low-mucin production (p<0.01 and p<0.05, respectively). In conclusions, this study firstly disclosed frequent GNAS mutations in IPNBs, similarly to IPMNs. This may suggest a common histopathogenesis of IPNBs and IPMNs. The status of KRAS mutations was inversely correlated to MUC2 expression and this may suggest heterogeneous properties of IPNBs. IPNBs with high-mucin production are characterized by perihilar location and high expression of MUC2 and MUC5AC, irrespective of the status of GNAS and KRAS mutations.

## Introduction

Intraductal papillary neoplasm of the bile duct (IPNB) is characterized by dilated intrahepatic bile ducts filled with noninvasive papillary or villous biliary neoplasm covering delicate fibrovascular stalks [1]. IPNB includes the previous categories of biliary papilloma and papillomatosis, and the term IPNB has been adopted in WHO classification 2010 [1]. IPNB is regarded as a biliary counterpart of intraductal papillary mucinous neoplasm of the pancreas (IPMN) and shows favorable prognosis [1]–[3]. In addition, IPNBs are not infrequently associated with invasive carcinoma (IPNB with an associated with invasive carcinoma) [1]. Some IPNBs show excessive mucin secretion and are described using the terms “mucin-producing bile duct tumor”, “mucin-hypersecreting bile duct tumor”, or “IPNB with macroscopically mucin-producing biliary tumor”[4]–[6]. This subset of IPNB is associated with hepatolithiasis [7] and has a tendency to show intestinal differentiation [4], [7], [8].

Guanine nucleotide-binding protein, α-stimulating activity polypeptide (GNAS) encodes the α-subunit of the stimulatory G-protein (Gαs), which mediates the regulation of adenylate cyclase activity through G-protein-coupled receptors. Activating mutations of GNAS at codon 201 have been detected in approximately two thirds of IPMNs of the pancreas [9], [10]. In addition, frequent GNAS mutations were reported in pituitary adenomas, colorectal villous adenomas and pyloric gland adenomas [11], [12]. In contrast to frequent mutation of GNAS in IPMNs, there have been few GNAS mutations in usual invasive ductal carcinoma of the pancreas (PDAC) [9], [10]. There have been only a few studies on GNAS mutation in cholangiocarcinomas and IPNBs, to our knowledge [13]–[15]. GNAS mutations were reportedly uncommon in IPNBs in a previous study [15]. GNAS mutation was not detected in cholangiocarcinomas and biliary intraepithelial neoplasia (BilIN) in our previous study [14]. v-Ki-ras2 Kirsten rat sarcoma viral oncogene homolog (KRAS) mutation is an early event within PanINs and occurs in up to 90% of early PanINs and in 95% pancreatic adenocarcinomas (PDACs)[16], [17]. KRAS mutation was detected in about a third of cholangiocarcinomas and BilINs [14].

Given frequent GNAS mutations in IPMNs, which are similar to IPNBs, we examined the status of GNAS and KRAS mutations. Furthermore, we analyzed their association with clinicopathological factors including the degree of mucin production and the expression of MUC mucin core proteins (MUC1, MUC2, MUC5AC, MUC5B and MUC6).

## Materials and Methods

### Classification of the biliary tree and IPNBs

The biliary tree is divided into intrahepatic, perihilar (the right, left and common hepatic ducts) and distal bile duct (extrahepatic bile ducts distal to the insertion of the cystic duct) [18]. The intrahepatic bile duct is classified as described previously [18]. IPNB were classified into perihilar and distal IPNB based on the location of tumor according to the TNM classification of malignant tumors [19].

### Preparation of human IPNB tissue specimens

We examined 29 patients with perihilar IPNB (12 non-invasive, 3 microinvasive and 14 invasive, M/F = 15/14) and 6 distal IPNB (all invasive M/F = 5/1). We defined the IPNB with microinvasion as IPNB with small foci of invasion in which the deepest invasion is limited to the mucosa in this study. The clinical and pathological features were summarized in Table. 1. Mucinous cystic neoplasms with ovarian-like stroma were excluded. Participants provided their written informed consent to participate in this study. The Ethics Committee of Kanazawa University approved this study and consent procedure. When the written consent was not obtained because of old samples, such samples were handled anonymously. The respective Ethics Committee also approved this. All of these specimens were obtained from the liver disease file of our department and affiliated hospitals. These specimens were fixed in 10% buffered formalin and embedded in paraffin, and more than 20 serial 3 µm thin sections were cut from each paraffin block. Some sections were stained for hematoxylin and eosin (HE) stain and the remaining were used for the following DNA extraction, mucin stain and immunostaining.

**Table 1:** Main clinical and pathological features in the patients examined.

	*Perihilar IPNB Non-invasive*	*Perihilar IPNB Invasive*	*Distal IPNB Invasive*
	(n = 15)	(n = 14)	(n = 6)
Age (mean ± SD, range)	63.1±13.1 (38–80)	64.3±9.2 (36–76)	66.6±15.0 (35–79)
Sex (M/F)	8/7	7/7	5/1
Location (L/R/CBD)	11/1/3	11/1/2	0/0/6
Tumor size (cm, mean ± SD, range)	3.3±2.2 (1.0–8.0)	4.8±1.8 (2.5–7.5)	3.5±1.9 (1.2–7.0)
Association of hepatolithiasis	3	11	0
Cyst formation	6	1	0
Type of differentiation (I/G/PB&O)	0/3/12	4/8/2	0/2/4
Degree of dysplasia (low/intermediate/high)	0/14/1	0/10/4	0/0/6
Invasion (no and micro-I/inv)	15/0	0/14	0/6
Histology of invasive carcinoma	///	Mucinous Ca: 6 Tubular Ca: 8	Tubular Ca: 6
Lymphnode metastasis (negative/positive)	15/0	11/3	5/1

IPNB, intraductal papillary biliary neoplasm; SD, standard deviation; M, male; F, female; L, left; R, right; CBD, common bile duct;

I, intestinal; G, gastric; PB, pancreatobiliary; O, oncocytic; micro-I, microinvasive; inv, invasive; Ca, carcinoma.

### Extraction of DNA samples and GNAS and KRAS mutation analysis

IPNBs and background livers were visualized and scraped off from 2 to 3 serial whole sections (3 µm) and DNA was isolated using the QIAMP DNA kit (QIAGEN). Approximately 10,000 cells were harvested from each lesion with and estimated tumour cellularity of >80%. Isolated DNA was then subjected to PCR amplification of the region of the GNAS gene coding codon 201 and KRAS gene containing codons 12 and 13. The forward and reverse primers for the GNAS codon 201 were 5′-ACTGTTTCGGTTGGCTTTGGTGA-3′ and 5′-AGGGACTGGGGTGAATGTC -AAGA-3′ and the primers for KRAS gene containing codons 12 and 13 were 5′-AGGCCTGCTGAAAATGACTG-3′ and 5′-ATCAAAGAATGGTCCTGCAC -3′, respectively. Amplifications were done by initial denaturation at 94°C for 3 min followed by 35 cycles of denaturation at 94°C for 1 min, annealing at 58°C for 1 min, and extension at 72°C for 1 min, followed by 10 min final extension at 72°C using TaqDNA polymerase (Takara EX Taq; Takara Bio). These PCR products were then purified using the QIAGEN PCR purification kit (QIAGEN) and sequenced by the Big Dye cyclic sequencing kit and ABI 310 sequencer (Applied Biosystems, Forster City, CA).

### Mucin stain and semiquantitative evaluation

**Figure 1:**
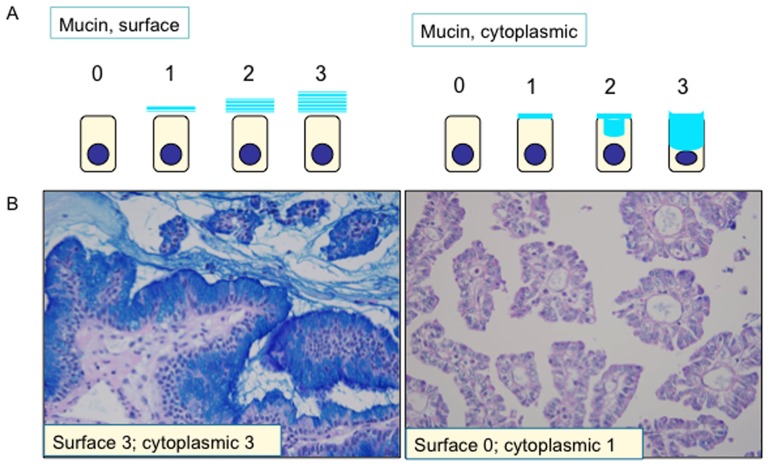
Semiquantitative evaluation of surface and intracellular mucin in intraductal papillary neoplasms of the bile duct (IPNBs). A) Schema of score 0–3 for surface and cytoplasmic mucin. B) Example of IPNBs with high- and low- mucin production. Left, an example of IPNB with high-mucin production; right, an example of IPNB with low-mucin production.

Each section was processed for double mucin stain with periodic acid Schiff stain after diastase-digestion and alcian blue (pH 2.5) (d-PAS/AB). Amount of mucin production was evaluated sumiquantitatively (score 0–3), being based on d-PAS/alcian blue positive mucin on the cell surface of tumor cells. Figure 1 demonstrates degrees of surface and intracellular mucin productions and showed examples.

### Immunostaining and semiquantitative evaluation

The expression of MUC1, MUC2, MUC5AC, MUC5B and MUC6 mucin was immunohistochemically assessed using a standard method as described previously [20]. Primary antibodies used in this study were shown in Table 2. A similar dilution of the control mouse IgG (Dako) was applied as negative control. The expression of MUC mucin core proteins (MUC1, MUC2, MUC5AC, MUC5B and MUC6) was evaluated according to the percentage of positive cells in each lesion: Score 0, less than 1%; score 1, 1–30%; score 2, 30–70%; score 3, more than 70%.

**Table 2:** Primary antibodies used in this study.

*Primary antibody*	*Type (clone)*	*Pre-treatment*	*dilution*	*Source*
MUC1	Mouse mono (DF3)	-	1: 50	Toray-Fuji bionics (Tokyo, Japan)
MUC2	Mouse mono (Ccp58)	MW-CB	1∶100	NovoCastra (New Castle, UK)
MUC5AC	Mouse mono (CLH2)	MW-CB	1∶200	NovoCastra (New Castle, UK)
MUC5B	Goat-poly	MW-CB	1∶50	Santa-Cruz (Santa Cruz, CA)
MUC6	Mouse mono (CLH5)	MW-CB	1∶200	NovoCastra (New Castle, UK)

mono, monoclonal antibody; poly, polyclonal antibody; MW, microwave treatment; CB, 0.05 M citrate buffer (pH 6);

### Statistical analysis

The Wilcoxon rank sum test and Kruscal-Wallis test with Dunn posttest was used in statistical analysis for the difference between 2 groups and among 3 or more groups, respectively. A *p* value <0.05 was considered significant. Correlation between 2 groups was assessed using Spearman's correlation test. A *p* value <0.05 was considered significant.

## Results

### Mutational analyses

#### GNAS mutation

**Figure 2:**
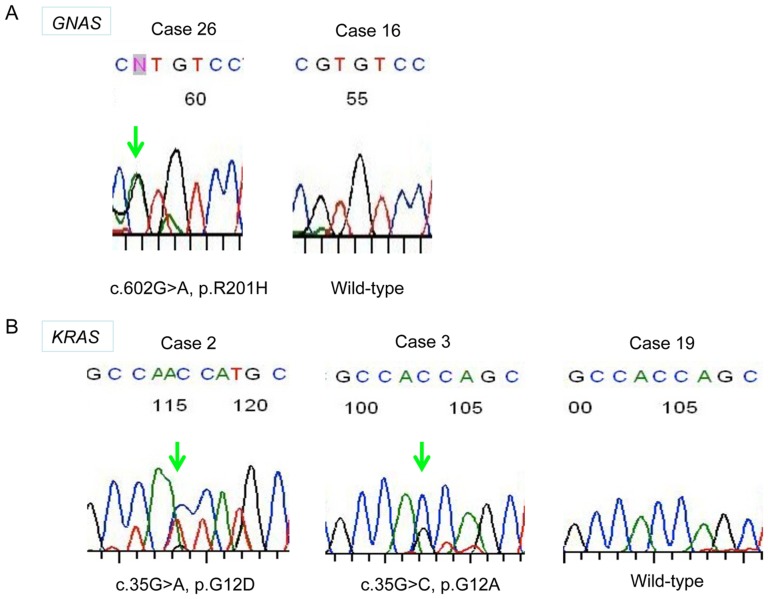
GNAS and KRAS mutations in intraductal papillary neoplasms of the bile duct (IPNBs). A) Representative sequencing trace of an IPNB without GNAS mutation (wild type, WT) and IPNBs with GNAS mutations. An arrow indicates miss-sense mutation. B) Representative sequencing trace of an IPNB without KRAS mutation (wild type, WT) and IPNBs with KRAS mutations. An arrow indicates miss-sense mutation.

Sequencing analysis was successfully performed in 30 gene samples extracted from the cases of IPNB. GNAS mutation was detected in 15 cases (50%) of 30 IPNBs, including 12 perihilar IPNBs (50%) and 3 distal IPNBs (50%)(Table 3). All GNAS mutation was at codon 201; cDNA 602G>A and cDNA 601C>A mutants were detected in 14 and 2 cases, respectively (Table 3, Figure 2A). One case harbored both cDNA 602G>A and cDNA 601C>A mutations. There was no significant difference between GNAS mutation and the degree of mucin secretion and other clinicopathological factors (Table 4).

**Table 3:** Clinicopatholoical features and the status of mutation in intraductal papillary neoplasms (IPNBs).

No.	Age	Sex	Site	Size (cm)	Dysplasia	Subtype	Invasion	Mucin	MUC2	MUC5AC	GNAS	KRAS
**1**	74	F	periH	8	Int	G	Micro-I	3	0	3	c.602G>A	c.35G>A
**2**	77	M	periH	2	Int	G	no	3	0	2	c.602G>A	c.35G>A
**3**	60	M	periH	3.3	Int	G	no	3	3	3	-	c.35G>A, C
**4**	59	M	periH	2	Int	G	no	3	0	1	c.602G>A	c.35G>A
**5**	59	F	periH	7.5	Int	I	Invasive	3	3	3	c.602G>A	-
**6**	63	M	periH	5	Int	G	Invasive	3	0	3	-	-
**7**	67	M	periH	5	Int	G	Invasive	3	1	2	c.602G>A	-
**8**	58	F	periH	2.5	Int	G/I	Invasive	3	3	3	-	-
**9**	62	M	periH	5	High	G/I	Invasive	3	2	3	-	-
**10**	38	F	periH	2.5	High	Onc	no	2	2	3	-	-
**11**	80	M	periH	7	Int	G	no	2	0	3	c.602G>A	-
**12**	49	M	periH	1.7	Int	PB	no	2	1	3	c.602G>A	-
**13**	78	F	periH	1.8	Int	G/pb	no	2	1	3	-	c.35G>A
**14**	72	F	periH	5	Int	G/pb	Invasive	2	2	3	c.601C>A	-
**15**	65	M	periH	7	High	pb/onc	Invasive	2	0	3	c.602G>A	-
**16**	68	F	periH	2.2	High	G/pb	Invasive	2	1	1	-	c.35G>A, C
**17**	74	M	distal	2.8	High	G/pb	Invasive	2	1	0	c.602G>A	-
**18**	57	M	periH	1	Int	G/pb	no	1	1	1	-	-
**19**	54	M	periH	7	Int	G	Micro-I	1	0	1	-	-
**20**	52	F	periH	2	Int	G/pb	Micro-I	1	0	3	-	NA
**21**	67	F	periH	3.2	Int	G/pb	no	1	0	1	c.601C>A c.602G>A	c.35G>A
**22**	77	F	periH	7	Int	G/pb	Invasive	1	0	3	-	NA
**23**	65	M	periH	2.5	Int	G/pb	Invasive	1	0	2	c.602G>A	c.35G>A
**24**	79	F	distal	2.5	High	pb	Invasive	1	0	2	c.602G>A	NA
**25**	77	M	distal	4	High	G/pb	Invasive	1	1	2	c.602G>A	NA
**26**	39	M	periH	6.5	High	pb	Invasive	0	0	0	c.602G>A	c.35G>A
**27**	76	F	periH	6	Int	G/pb	Invasive	0	0	3	-	c.35G>A
**28**	35	M	distal	7	High	pb	Invasive	0	0	2	-	-
**29**	72	M	distal	1.2	High	pb	Invasive	0	0	1	-	c.35G>A
**30**	64	M	distal	3.5	High	pb	Invasive	0	0	1	-	c.35G>A

M, male; F, female; Peri H, perihilar; Int, intermediate; I, intestinal; G, gastric; PB, pancreatobiliary; Onc, oncocytic; micro-I, microinvasion; NA, not available.

**Table 4:** Correlation between the status of GNAS and KRAS mutations and clinical and pathological factors.

	*GNAS mutation*	*KRAS mutation*
Location	ns	ns
Size	ns	ns
Invasion	ns	ns
Lymph node metastasis	ns	ns
Degree of dysplasia	ns	ns
Mucin production	ns	ns
MUC1	ns	ns
MUC2	ns	p<0.05
MUC5AC	ns	ns
MUC6	ns	ns
MUC5B	ns	ns

ns, no significance.

#### KRAS mutation

Sequencing analysis was successfully performed in 26 gene samples extracted from the cases of IPNB. Sequencing analysis for KRAS was failed in 4 gene samples for unknown reason. KRAS mutation was detected in 12 cases (46.2%) including 10 perihilar IPNB (45.5%) and 2 distal IPNBs (50%)(Table 3). Ten cases with KRAS mutation showed GGT to GAT at codon 12 and 2 cases harbored both GGT to GAT and GGT to GCT at codon 12 (Figure 2B). KRAS mutation was significantly inversely correlated with MUC2 expression (Table 4). There was no significant correlation between GNAS and KRAS mutations.

### Mucin production in IPNBs

**Figure 3:**
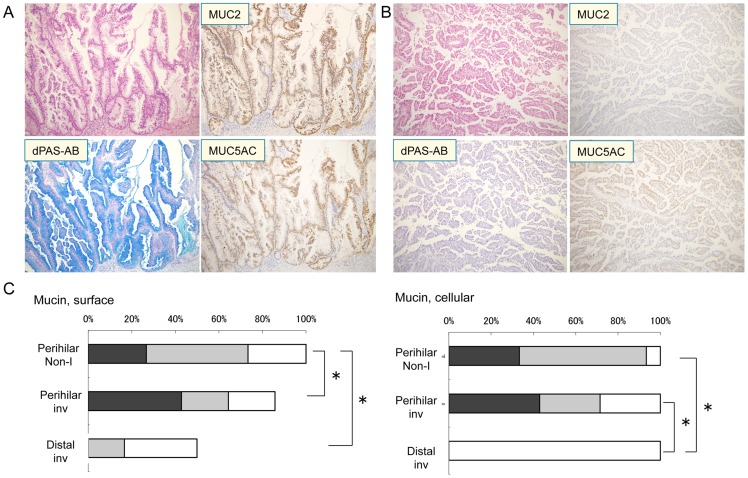
Intraductal papillary neoplasms of the bile duct (IPNBs) with high- and low- mucin production and the expression profiles of MUC mucin core protein. A) An example of IPNB with high- mucin production. IPNB with high- mucin production is composed of tall columnar tumor cells showing abundant mucin production in double mucin stain with periodic acid Schiff stain after diastase-digestion and alcian blue (pH2.5) (d-PAS/AB) (scores; surface 3, cytoplasmic 3). The tumor cells show extensive immunoreactivity for MUC2 and MUC5AC. B) An example of IPNBs with low-mucin production. IPNB with low-mucin production is composed of cuboidal tumor cells showing less mucin production (scores; surface 0, cytoplasmic 1). The tumor cells show no immunoreactivity for MUC2 and focal immunoreactivity for MUC5AC. Hematoxylin and eosin, d-PAS/AB and the immunostaining for MUC2 and MUC5AC and hematoxylin. x200. C) Semiquantitative evaluation of the degree of mucin production in perihilar IPNBs with and without invasion and distal IPNBs (all with invasion). White column, score 1; half-tone column, score 2; black column, score 3. *, p<0.05. Non-I, without invasion; inv, with invasion.

All IPNBs showed mucin production to various degrees. Twenty and 15 patients were divided into high- and low- mucin production, respectively. Representative histology of IPNBs with high- and low-mucin production was shown in Figure 3A and 3B. The degree of mucin production was significantly higher in perihilar IPNBs than distal IPNBs (p<0.05). (Figure 3C).

**Figure 4:**
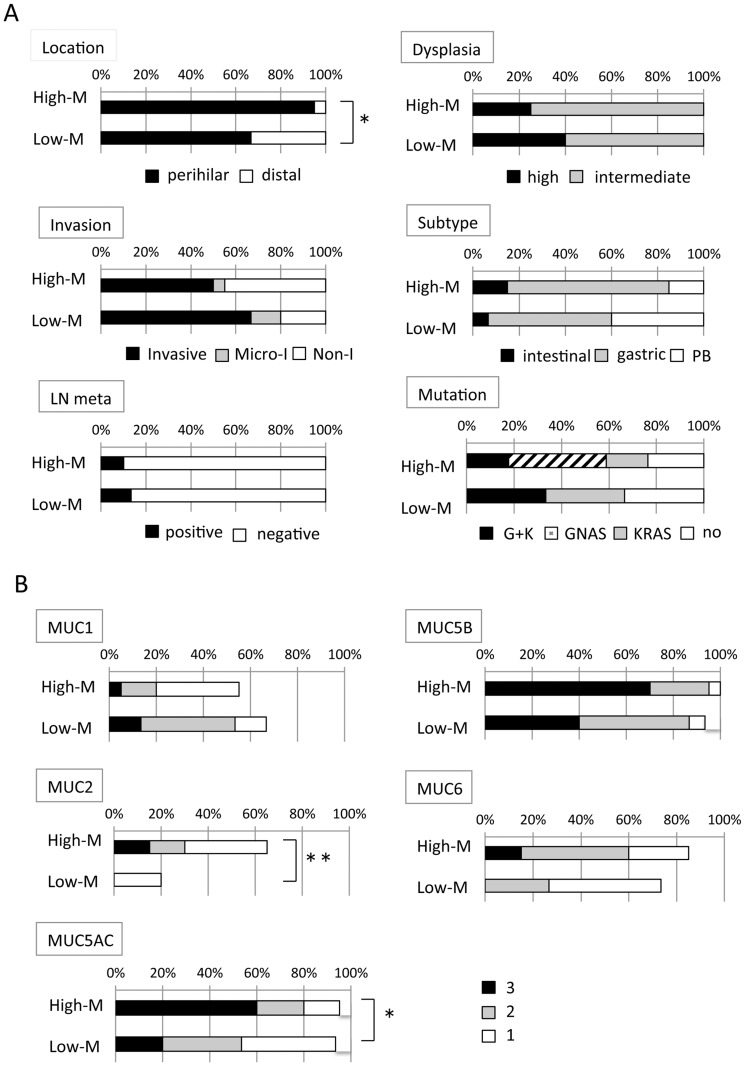
Clinicopathological factors and MUC mucin expression profiles in IPNBs with high- and low- mucin production. A) Clinicopathological factors in IPNBs with high- and low-mucin production. *, p<0.05. Micro-I, microinvasive; Non-I, without invasion; PB, pancreaticobiliary; G+K, both GNAS and KRAS mutations. B) MUC mucin expression profiles in IPNBs with high- and low-mucin production. *, p<0.05; **, p<0.01.

Correlation of the degree of mucin production with clinicopathological factors including the expression profiles of MUC mucin core proteins was analyzed (Figure 4). Invasion tended to be associated with low-mucin production, although there was no significance. Regarding correlation between the degree of mucin production and the status of GNAS and KRAS mutations, it is noted that all IPNBs with only GNAS mutation (n = 7) were included in the group of high-mucin production (Figure 4A and Table 3). On the other hand, there was no IPNB with only GNAS mutation in the group of low-mucin production (Figure 4A). MUC2 and MUC5AC expression was significantly higher in IPNBs with high-mucin production, than those with low-mucin production (p<0.01 and p<0.05, respectively) (Figure 4). The expression of MUC1 was significantly correlated with invasion, but it was not significantly associated with degree of mucin production.

Most IPNBs with high mucin production were intestinal or gastric type, whereas some IPNBs with low-mucin production were pancreaticobiliary and oncocytic types (Figure 4). The degree of dysplasia tended to be higher in IPNB with low-mucin production than IPNBs with high-mucin production, although there was no significant difference.

## Discussion

The findings obtained in this study are summarized as follows: 1) GNAS mutations were detected in a half of IPNBs, irrespective of clinicopathological factors; 2) KRAS mutations were detected in about a half of IPNBs and the status of KRAS mutations was inversely correlated with the degree of MUC2 expression; 3) IPNBs with high-mucin production was characterized by perihilar location and high expression of MUC2 and MUC5AC. 4) All IPNBs with GNAS mutations only were high-mucin production IPNBs.

This study firstly disclosed rather high prevalence of GNAS mutations in IPNBs. GNAS mutations were detected in a half of IPNBs, irrespective of mucin production, phenotypes and the degree of dysplasia in this study. There has been only one study regarding the status of GNAS mutations in IPNBs, in which low frequency (3%) of GNAS mutations in IPNBs was reported, to our knowledge [15]. The mutation sites in GNAS in IPNBs identified in this study are at codon 201 (R201C and R201H) which are common activating mutations in IPMNs and other tumors [9], [10]. Embryological and anatomical similarities of perihilar biliary tract and pancreas are well known and similarities of biliary tract and pancreatic diseases have been suggested [3]. For example, preneoplastic or early intraepithelial neoplasms of the biliary tract, such as BilIN and IPNBs, show similar morphological or genetic changes to their pancreatic counterparts, such as PanINs and IPMNs [3]. Therefore, rather high prevalence of GNAS mutations in IPNBs similar to those in IPMNs may be one of evidence suggesting the similarities of IPNBs to IPMNs.

In this study, there was no association between the status of GNAS mutations and any clinicopathological factors. Similarly, mutations in *GNAS, KRAS,* or both genes did not appear to be associated with any of the clinicopathological features of IPMN (sex, age, morphologic variation, et. al.) in previous study [9]. Therefore, GNAS mutation itself may not be linked to any specific features such as mucin production and phenotypes in both IPNBs and IPMNs. However, it is of interest that all IPNBs with only GNAS mutations were IPNBs with high-mucin production. In contrast, no IPNBs with only GNAS mutations were low-mucin production. This finding suggests that the presence of GNAS mutations may be associated with high-mucin production in IPNBs.

Interestingly, GNAS mutations are frequent in IPNBs in this study, whereas GNAS mutations were not detected in all ICCs and BilINs in our previous study [14]. Similarly, GNAS mutations are found frequently in IPMNs, whereas they are absent to rare in usual pancreatic ductal adenocarcinomas (PDACs) [9], [10]. Therefore, it is conceivable that GNAS mutations may play a driving role in the development of papillary tumor in bile ducts and pancreas in common. In contrast, it is conceivable that GNAS mutations may not participate in the carcinogenesis pathway via flat precursor lesion and usual ICCs and PDACs in biliary tracts and pancreas in common. In a recent study, GNAS mutations were detected in 9.3% of liver fluke-associated CC [13]. These CCs with GNAS mutations may be derived from via papillary precursor lesions; IPNBs.

There has been only one study regarding the status of GNAS mutations in IPNBs, to our knowledge [15]. In the previous paper, the frequency of GNAS mutation was low (3%). The authors discussed the difference between IPNBs and IPMNs of the pancreas in which GNAS mutation was frequent. Different from this previous study, GNAS mutations were common in the present our study. Although reasons for this discrepancy are unknown, one possible reason may be difference in location of IPNBs. Most IPNBs in our study were at perihilar regions and intracystic papillary neoplasms in gallbladder were not included, whereas IPNBs were mainly located extrahepatically or in the gallbladder in the previous study [15]. It is well known that there are differences in properties between neoplasms in gallbladder and neoplasms in bile duct. For example, the overexpression of p53 was more frequent in gallbladder carcinomas (61.5%) as compared to ICCs (18.2%) and extrahepatic CCs (38.1%) [14]. So, it is natural that the status of GNAS mutations is different between perihilar IPNBs and intracystic papillary neoplasms in gallbladder. In fact, only one IPNB with GNAS mutation detected in the previous study was a multifocal intestinal subtype intrahepatic IPNB with high-grade dysplasia [15]. Another possible reason could be the heterogeneity of the IPNBs samples, as heterogeneity in IPMNs have been reported with regard to GNAS mutations [10], [15]. Other possibilities for this difference may be related to differences of in race, epidemiology, pathogenesis, difference of phenotypes of IPNBs in each study. In addition, the method to detect GNAS and KRAS mutation was different between this study and previous studies [10], [15].

GNAS mutation may be related to the initiation of tumor genesis and represent indolent villous/papillary growth pattern of tumors in various organs. It is of interest that GNAS mutations were frequently detected in benign tumors such as colorectal villous adenoma (83%) [11], appendiceal low- grade mucinous neoplasm (50%) [21], pyloric gland adenoma (63%) [12], inflammatory type hepatocellular adenoma (5%)[22] and pituitary adenoma (40%)[23]. The mutation sites in GNAS are at codon 201 (R201C and R201H) in these tumors and these mutations are known to cause disruption of the intrinsic hydrolytic activity of Gsα, which results in constitutive activation of its function [24]. *GNAS* mutations were observed in low-grade tumors as well as in high-grade tumors and invasive tumors in IPNBs and IPMNs [9]. These results suggest that *GNAS* mutations may be associated more with initiation but less with progression of the neoplasm with some specific clinicopathological features.

KRAS mutations were detected in 46.2% of IPNBs in the present study. Either GNAS or KRAS mutations were identified in 73.1% in IPNBs. In previous studies, the prevalence of KRAS mutations was reportedly 17.6% [15] to 28.6% [25] of IPNBs. Although reasons for these differences remain to be unknown, the prevalence of KRAS mutations in our study tends to be higher than that in previous reports. KRAS mutation was detected in one third of ICCs and also one third of BilINs associated with hepatolithiasis in our recent study, suggesting that early step of carcinogenesis may already occur in this region [14]. So, the frequency of KRAS mutations may be higher in IPNBs than usual ICCs arising via flat precursor lesions (BilINs). Frequent KRAS mutations were also reported in IPMNs (48% to 81%) [9], [10].

Two thirds of IPNBs with KRAS mutations harbored concomitantly GNAS mutations in this study, although there was no significant association between the status of GNAS and KRAS mutations. A significant proportion (25% to 51%) of IPMNs showed concurrent mutations in GNAS and KRAS [9], [10]. Similarly, frequent concurrent presence of KRAS and GNAS mutations was also detected in colorectal villous adenomas [11] and pyloric gland adenomas [12]. Taken together, the simultaneous activation of *GNAS*- and *KRAS*–dependent pathways might cooperatively promote tumor genesis in various gastrointestinal organs.

It is of interest that there was an inverse correlation between the status of KRAS mutation and MUC2 expression in the present study. The degree of mucin production also tended to be inversely correlated to the status of KRAS mutations, although there was no significant correlation. In a previous study, no significant differences between the IPNBs with and without KRAS mutations for patient characteristics and clinical and pathologic features (age, gender, location, et al.)[15]. The direct relationship between the status of KRAS mutation and MUC2 expression remains to be elucidated at this moment. Since KRAS mutation is common in BilIN, PanIN and PDACs in which MUC2 expression is rare, KRAS mutations and MUC2 expression may suggest different pathways in the carcinogenesis. Although no significant correlation was found in this study, GNAS mutation might be more associated with intestinal differentiation. Interestingly, the introduction of the mutant GNAS into a colorectal cancer cell line markedly induced MUC2 and MUC5AC expression, but did not promote cell growth either in vitro or in vivo [21].

In the present study, all IPNBs showed mucin production to various degrees. MUC2 and MUC5AC expression was significantly high in IPNBs with high-mucin production, compared to those with low-mucin production. The association of mucin hypersecretion and MUC2 mucin expression agreed to a previous report [4]. Invasion tended to be associated with low-mucin production, although there was no significance. The expression of MUC1 was significantly correlated with invasion in our previous study [8], but it was not significantly associated with degree of mucin production in this study. Most IPNBs with high mucin production were intestinal or gastric type, whereas IPNBs with low-mucin production tended to be pancreaticobiliary and oncocytic types. The degree of dysplasia tended to be high in IPNBs with low-mucin production, compared to IPNBs with high mucin production, although there was no significant difference.

It is conceivable that properties of MUC2 and MUC5AC mucin may be regarded as high-mucin production showing viscous mucin on epithelial cell surface. Mucus forms a protective and selective barrier as well as a lubricating film over wet epithelial surfaces in the mammalian body, including those of the respiratory, ocular, reproductive, and gastrointestinal (GI) systems [26], [27]. MUC2, MUC5AC and MUC6 are known as intestinal, gastric surface (foveolar) and pyloric gland type mucins, respectively [26], [27]. MUC2 mucin is a gel-forming mucin expressed in colon and its viscosity is high. MUC2 mucin forms trimerization in MUC2-rich polymeric mucin and these trimers are the fundamental units of a porous, lamellar network [28].MUC5AC is also a gel-forming mucin expressed in gastric surface epithelial cells (foveolar cells) and shows sticky and viscous feature. The other common secreted mucins, MUC5AC, MUC5B, and MUC6, are considered to retain a linear morphology, and no evidence for trigonal formation has been reported for these mucins [29]. In contrast, physiological biliary mucin such as MUC3 is a membrane-binding mucin. MUC5B is a gel-forming mucin, but the viscosity is lesser than MUC2 and MUC5AC. Therefore, the properties of MUC2 and MUC5AC mucin expressed in IPNBs may be responsible for findings regarded a high-mucin production.

In conclusion, this study firstly highlights an involvement of GNAS mutations as well as KRAS mutations in IPNBs, similarly to IPMNs, suggesting a common pathogenesis of IPNBs and IPMNs. IPNB with high mucin secretion was associated with perihilar location and the expression of MUC2 and MUC5C expression.
